# Unilateral biportal endoscopy for upper lumbar disc herniation: surgical challenges and clinical outcomes

**DOI:** 10.20452/wiitm.2025.17977

**Published:** 2025-09-08

**Authors:** Jiashen Shao, Zhiwu Zhang, Hai Meng, Jisheng Lin, Zihan Fan, Qi Fei

**Affiliations:** Department of Orthopedics Beijing Friendship Hospital Capital Medical University Beijing China

**Keywords:** clinical outcomes, percutaneous endoscopy, unilateral biportal endoscopy, upper lumbar disc herniation

## Abstract

**INTRODUCTION:**

Upper lumbar disc herniation (ULDH) is a rare type of herniation occurring at the L1/L2 or L2/L3 level. Due to its high location, associated with small spinal canal volume and complex anatomy, surgical treatment of this condition is challenging.

**AIM:**

The aim of this study was to evaluate the clinical efficacy of unilateral biportal endoscopy (UBE) in the treatment of ULDH, and to assess short-term clinical outcomes.

**MATERIALS AND METHODS:**

This was a retrospective analysis of patients with L1/L2 or L2/L3 LDH who underwent UBE decompression surgery between June 2021 and June 2024 at the Department of Orthopedics of the Beijing Friendship Hospital. Demographic characteristics and surgical data were analyzed, and the Oswestry Disability Index (ODI), Japanese Orthopaedic Association (JOA), and visual analog scale (VAS) scores for lower back and leg pain were collected from the patients preoperatively, postoperatively, and at the final follow-up. Clinical improvement was assessed using the MacNab criteria.

**RESULT:**

During the study period, a total of 18 patients with ULDH who met the inclusion criteria underwent UBE decompression surgery. The mean (SD) follow-up was 17.5 (9.2) months. Postoperative VAS, JOA, and ODI scores showed significant improvement, as compared with the preoperative values, whereas the values obtained at the final follow-up did not differ significantly from those recorded in the early postoperative period. Only 1 patient showed poor improvement and developed postoperative complications.

**CONCLUSION:**

UBE technology has significant potential in the field of ULDH treatment; however, further large-scale clinical trials are warranted to confirm its long-term efficacy.

## INTRODUCTION

Lumbar disc herniation (LDH) is a spinal condition commonly encountered in clinical practice, often characterized by persistent low back pain, radicular pain in the lower limbs, and even motor dysfunction, significantly impacting patient quality of life.^1^;^2^ While LDH is highly prevalent, upper LDH (ULDH) is relatively uncommon, constituting only a small proportion of cases.^3^;^4^;^5^ The definition of ULDH is controversial; the most widely accepted one is herniation occurring at the L1/L2 or L2/L3 level.^4^;^5^ Because of its higher anatomical location, smaller spinal canal volume, and complex anatomical structure of the upper spine, ULDH poses significant challenges for surgical treatment. Currently, research on ULDH is limited, and its treatment primarily relies on traditional open surgeries, which are associated with considerable surgical trauma, prolonged recovery period, and a high risk of perioperative complications. Therefore, exploring a less invasive and efficient treatment modality has become an urgent clinical need.

Unilateral biportal endoscopy (UBE) is an emerging minimally-invasive technique that has garnered considerable attention among spinal surgeons in recent years.^6^;^7^;^8^ In comparison with traditional open surgery, UBE offers distinct advantages, including minimal invasiveness, faster recovery, a wider operative field, and superior visualization. It has demonstrated excellent outcomes in the treatment of LDH.^9^ Through a minimally-invasive dual-port approach, UBE avoids extensive soft tissue dissection commonly required in traditional open surgeries, enhancing surgical precision, flexibility, and safety. Thus, it is a promising treatment option for ULDH that could reduce perioperative risks and accelerate postoperative recovery.

Although the UBE technique has demonstrated great potential in the field of spinal surgery, research on its application in ULDH treatment is still limited.^10^ Exploring the efficacy of UBE in various types of lumbar degenerative diseases could help expand the range of indications for this technology.

## AIM

The aim of this study was to evaluate the application of UBE technology in the treatment of ULDH, focusing on its potential advantages in terms of surgical safety, efficacy, recovery time, and occurrence of complications.

## MATERIALS AND METHODS

### Patient selection

We performed a retrospective analysis of patients with LDH at the L1/L2 or L2/L3 level who underwent UBE decompression surgery between June 2021 and June 2024 at the Department of Orthopedics, Beijing Friendship Hospital, Beijing, China. Inclusion criteria were: 1) age of at least 18 years; 2) presence of ULDH (at the L1/L2 or L2/L3 level) confirmed on imaging, without concomitant intervertebral instability (usually patients with a visual analog scale [VAS] score <⁠6 for lumbar pain); and 3) treatment with UBE. Exclusion criteria comprised: 1) age below 18 years; 2) a history of lumbar spine surgery, including lumbar decompression surgery or lumbar interbody fusion at the same level; and 3) presence of lumbar spinal stenosis, spinal infection, tumor, or tuberculosis.

### Surgical technique

The clinical symptoms of ULDH are often atypical. Therefore, when it coincides with herniation or spinal stenosis in other lumbar segments, further confirmation of the responsible segment by selective nerve root blocking (SNRB) is often required preoperatively.^11^ In our institution, this procedure is usually performed in the operating room.

The patient was placed prone on the operating Table and oblique plain films were taken to confirm the puncture site. After local anesthesia with 2% lidocaine, the spinal puncture needle was advanced under fluoroscopic guidance until the patient experienced a stinging sensation in the lower extremities. Subsequently, a contrast medium (Iohexol, 300 mg/ml; GE Healthcare, Cork, Ireland) was injected to confirm the position of the nerve root at the injection site. Then, 0.5 ml of 0.25% bupivacaine diluted in saline was injected into the periphery of the nerve root via the same route.

**Figure 1:**
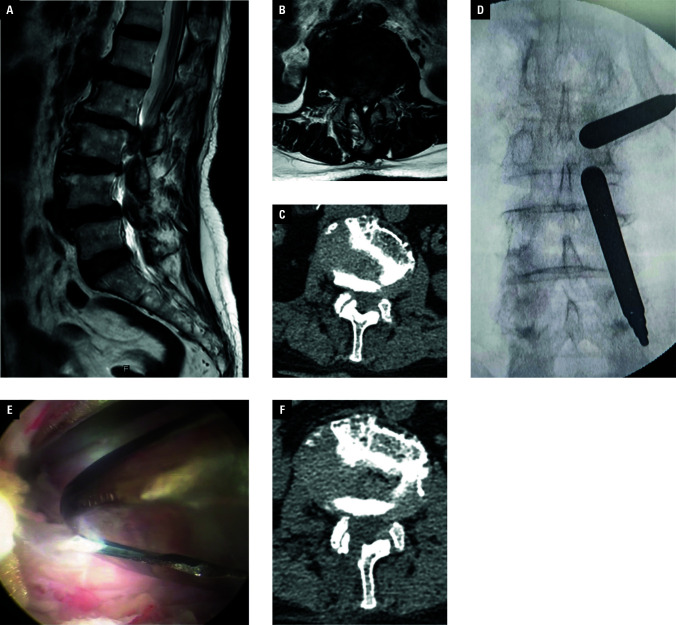
Imaging findings in a 75-year-old woman who underwent unilateral biportal endoscopy for L2/L3 disc prolapse with spinal stenosis; **A**–**C** – magnetic resonance imaging and computed tomography (CT) scans showing L2/L3 disc herniation (arrows); **D** – intraoperative fluoroscopy showing successful establishment of a surgical tunnel for an interlaminar approach; **E** – intraoperative decompression and removal of the prolapsed nucleus pulposus followed by nerve root release; **F** – postoperative CT image suggesting adequate decompression

The process of preoperative preparation, including surgical segment determination and surgical access creation, is described in our previous study^6^ Figure 1. The soft tissues on the surface of the intervertebral space were processed under a radiofrequency probe (BONSS, Jiangsu, China), enabling gradual exposure of the lower edge of the upper lamina, the base of the spinous process, the articular synchondrosis, and the upper edge of the lower lamina. Part of the bone (including part of the lower edge of the lamina, the upper edge of the lamina, and the articular process) was removed with a grinder, osteotome, and vertebral occluder. After exposing the beginning of the ipsilateral ligamentum flavum, a nerve dissector and probe hook were used to release its attachment points from the lamina. Probing was performed to determine whether there were adhesions between the ligament and the dural sac. Subsequently, the ligamentum flavum was resected to expose the dural sac and nerve roots. After exploring and separating the adhesions between the nucleus pulposus and the dural sac, the protruding nucleus pulposus was gradually removed using forceps. Once complete decompression was confirmed, hemostasis was performed using a radiofrequency probe. The incisions were then closed, and drains were placed.

### Data collection and analysis

Patient data were obtained from the electronic medical record system of the Beijing Friendship Hospital. Demographic characteristics and surgical data collected for analysis included age, sex, body mass index, comorbidities, duration of symptoms, operative time, length of hospital stay, level of surgical intervention, American Society of Anaesthesiologists classification, total blood loss, and postoperative complications. Oswestry Disability Index (ODI), Japanese Orthopaedic Association (JOA), and VAS scores were collected from patients preoperatively, postoperatively, and at the final follow-up. Time points of follow-up were set at 3, 6, 9, and 12 months postsurgery. Thereafter, follow-up was scheduled every 6 months. The final follow-up assessment was conducted prior to the completion of this study. Two researchers (JS and ZZ) were responsible for data extraction and follow-up, while a third researcher (HM) checked the accuracy of the data. All data were entered into a Microsoft Excel spreadsheet (2013; Microsoft Corp., Redmond, Washington, United States) for consistency checking and data cleaning.

### Statistical analysis

The R studio 4.4.0 software (R Foundation for Statistica Computing, Vienna, Austria) was used for statistical calculations. The normality of distribution for continuous variables was assessed using the Shapiro–Wilk test. Based on the results, the data were expressed as mean (SD) or median (interquartile range [IQR]). Categorical data were expressed as frequencies and percentages. Repeated measures data were analyzed using the analysis of variance with repeated measures design, and individual effects were further analyzed after the presence of an interaction, with multiple comparisons corrected using the Bonferroni correction. The *F* value represents the ratio of the variance between groups to the variance within the groups, reflecting the magnitude of intergroup variation relative to intragroup variation. A smaller *F* value indicates less variation between the groups, while a larger *F* value suggests greater variation between the groups. All statistical tests were 2-tailed, with a *P* value below 0.05 considered significant.

### Ethics statement

The experimental protocol was reviewed and approved by the Ethics Committee of the Beijing Friendship Hospital (2022KY087). The study was conducted in accordance with the experimental protocol and the Declaration of Helsinki, and informed consent was obtained from all participants. Apart from routine treatment during hospitalization, the patients did not receive any other treatments relevant to this study, nor were they exposed to any additional risks.

## RESULTS

### General characteristics

Between June 2021 and June 2024, a total of 18 patients (6 men and 12 women) with ULDH who met the inclusion criteria underwent UBE decompression surgery at our institution. Mean (SD) patient age was 54.8 (8.2) years, and median (IQR) duration of symptoms was 55.2 (3–111) months. Mean (SD) follow-up was 17.5 (9.2) months, mean (SD) operative time was 139.8 (59.2) minutes, and the patients were hospitalized for a mean (SD) of 11.8 (6.9) days. Ten patients (55.6%) had coexisting spinal stenosis, and 5 (27.8%) underwent preoperative diagnostic SNRB. Detailed demographic characteristics and perioperative outcomes are shown in Table 1.

**Table 1:** Demographic and surgical characteristics of the study group (n = 18)

Parameter		Value
Age, y		54.8 (8.2)
Sex	Men	6 (33.3)
	Women	12 (66.7)
BMI, kg/m^2^		25.1 (3.7)
Comorbidities		8 (44.4)
Duration of symptoms, mo, median (IQR)		55.2 (3–111)
ASA class	I	10 (55.6)
	II	6 (33.3)
	III	2 (11.1)
Operated segment	L1/L2	2 (11.1)
	L2/L3	16 (88.9)
Operative time, min		139.8 (59.2)
Estimated blood loss, ml		51.7 (19.8)
Length of hospital stay, d		11.8 (6.9)
Complications		1 (5.6)
Surgical outcomes according to the MacNab criteria	Excellent	5 (27.8)
	Good	8 (44.4)
	Fair	4 (22.2)
	Poor	1 (5.6)
Follow-up, mo		17.5 (9.2)

Values are presented as mean (SD) or number (percentage) unless indicated otherwise.

Abbreviations: ASA, American Society of Anesthesiologists; BMI, body mass index; IQR, interquartile range

### Clinical outcomes

**Figure 2:**
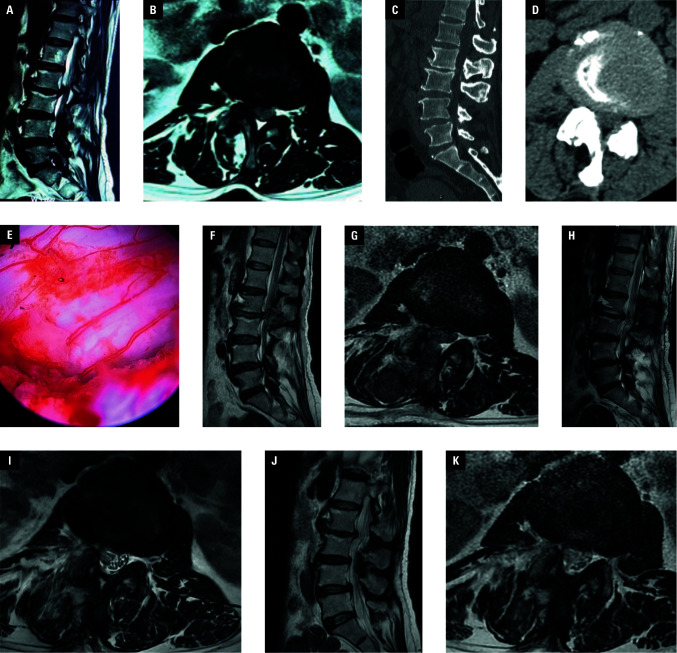
Schematic representation of changes in values of assessment indicators at different follow-up time points. Boxes represent interquartile ranges. Horizontal lines in the middle of the boxes represent the median. Vertical lines extending upwards and downwards from each box represent the distribution range of nonoutliers. Dots represent each sample.

Abbreviations: see Table 2

According to the MacNab criteria, 5 patients (27.7%) showed excellent improvement, 8 (44.4%) experienced good improvement, 4 (22.2%) showed fair improvement, and 1 individual (5.6%) had poor improvement. The patient with a poor score underwent postoperative posterior lumbar decompression and hematoma removal. The mean (SD) VAS value for postoperative low back pain was 2.9 (0.8), showing a marked improvement compared with the values recorded in the preoperative period (*P* = 0.007). At the final follow-up, the mean (SD) value decreased to 2.5 (1.2), as compared with the postoperative score, but the difference was not significant. Similarly, the mean (SD) VAS score for leg pain improved significantly from 5.3 (1.7) before surgery to 2.2 (1.2) postprocedure, and decreased further to 2.1 (0.8) at the final follow-up, but the difference was no longer significant. With respect to the ODI scores, the mean (SD) values decreased from 50.1 (12) before surgery to 18.2 (13.7) postoperatively (*P* <⁠0.001). At the final follow-up, the mean (SD) ODI score was 21.4 (8.3), which was not significantly different from the postoperative score. The mean (SD) pre- and postoperative JOA scores were 16 (2) and 23.8 (3.3), respectively (*P* <⁠0.001). At the final follow-up, the mean (SD) JOA score decreased slightly as compared with the postoperative value, but the difference was not significant (Table 2 and Figure 2).

**Table 2:** Pre- and postoperative clinical outcomes of the study group

Characteristics	Preoperative	Postoperative	Final follow-up	*F* value	*P* value
ODI score, %	50.1 (12)	18.2 (13.7)	21.4 (8.3)	41.38	<⁠0.001
JOA score, points	16 (2)	23.8 (3.3)	22.4 (2.3)	46	<⁠0.001
VAS for lower back pain, points	4.3 (1.4)	2.9 (0.8)	2.5 (1.2)	13.33	<⁠0.001
VAS for leg pain, points	5.3 (1.7)	2.2 (1.2)	2.1 (0.8)	36.11	<⁠0.001

Values are presented as mean (SD).

Abbreviations: JOA, Japanese Orthopaedic Association; ODI, Oswestry Disability Index; VAS, visual analog scale

### Complications and management

**Figure 3:**
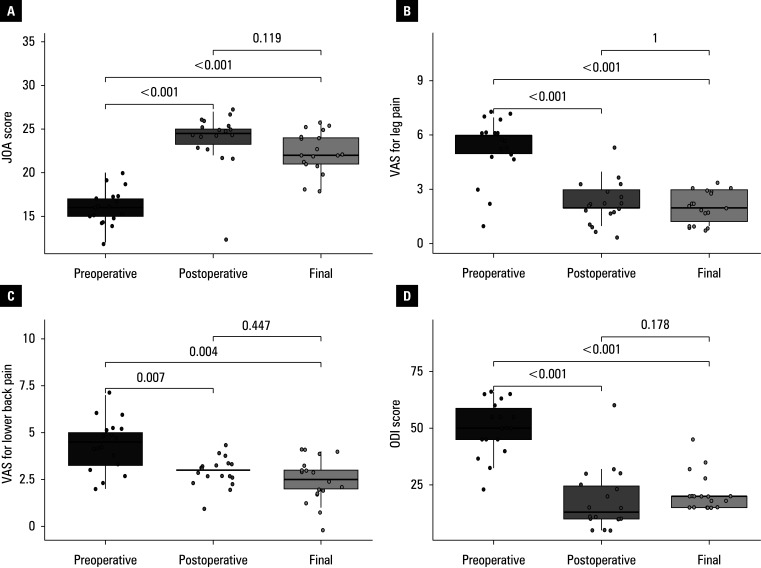
Imaging findings in a 56-year-old woman who underwent postoperative spinal epidural hematoma (PSEH) removal following unilateral biportal endoscopic (UBE) spinal surgery; **A**–**D** – pre-UBE computed tomography and magnetic resonance imaging (MRI) showing a herniated L2/L3 intervertebral disc with spinal stenosis; **E** – endoscopic image during UBE surgery showing dural injury (arrow); **F**, **G** – MRI after L2/L3 laminectomy showing PSEH with neurological deficit (lower limb weakness); **H**, **I** – MRI after PSEH removal showing substantial clearance of the hematoma; **J**, **K** – MRI 3 months after PSEH removal showing no recurrence of the hematoma

A single patient suffered an injury to the cauda equina and dural sac due to intraoperative entrapment of bone fragments Figure 3. She experienced postoperative numbness in the perineum and lower extremities, along with decreased muscle strength in the lower extremities. Postoperative magnetic resonance imaging indicated hematoma formation at the surgical site. Posterior lumbar open surgery for laminar decompression and hematoma removal was performed 4 days after the primary intervention. The patient’s symptoms improved after surgery. She was subsequently discharged from the hospital and referred for rehabilitation.

## DISCUSSION

ULDH is a rare condition, and studies focusing on its treatment remain limited. Consequently, there is a lack of substantial evidence to serve as a reference for evaluating the effectiveness and safety of UBE in treating this specific subset of LDH patients. The rarity of ULDH, coupled with its unique anatomical and pathological characteristics, poses significant challenges for surgical intervention, making it an area of particular interest among spinal surgeons.^3^;^12^;^13^

Application of UBE for the treatment of ULDH is technically challenging. The small volume of the spinal canal at higher lumbar levels greatly limits the workspace and increases the complexity of surgical manipulation. In addition, the narrow space in this region and closeness of surgical instruments to the dura and nerve roots during the operation increase the risk of complications, such as nerve root or dural injuries.^12^;^13^ These factors not only demand increased surgical precision but also contribute to prolonged operative times, as compared with surgeries performed at lower lumbar levels. The minimally-invasive approach and enhanced visualization facilitated by UBE could help overcome these challenges, highlighting the need for further investigation into its efficacy and safety in this context.

The application of UBE for the treatment of ULDH has shown several advantages in the perioperative stage, such as facilitation of rapid recovery.^14^;^15^ The minimally-invasive nature of the UBE technique allows for reducing soft tissue injury, preserving the integrity of the paraspinal muscle tissues, and enables patients to achieve functional recovery faster than with conventional open surgery. In addition, UBE allows for precise surgical vision and controlled soft tissue dissection, resulting in significantly less intraoperative blood loss. The average intraoperative blood loss in our study was only 51.7 ml, which is markedly lower than in open surgery.

Another major advantage of UBE is reduced operative time. Using 2 operating ports, the spine surgeon is provided with a clear and well-defined surgical area, allowing for more efficient and precise management of the lesion. Moreover, compared with open surgery, UBE also significantly reduces the incidence of perioperative complications, such as nerve root or dural injury.^16^;^17^ The real-time guidance provided by the endoscope facilitates delicate dissection and minimizes the risk of accidental nerve or dural injury, which is particularly important in the high lumbar spine, where the anatomy is relatively complicated.

Notably, in our center, SNRB is performed preoperatively in many of the patients with ULDH. In comparison with low-level LDH patients, these individuals usually have atypical clinical symptoms, manifesting as chronic and persistent low back pain, anterior thigh pain, and, infrequently, as sudden onset cauda equina syndrome. Therefore, accurate preoperative diagnosis is important for patients with ULDH, particularly those with coexisting lower LDH, in whom preoperative SNRB is a favorable method of clarifying the responsible segment.

In terms of short-term clinical outcomes, UBE demonstrated favorable results within 6 months of surgery. Postoperative clinical outcomes, evaluated using the MacNab criteria and ODI, JOA, and VAS scores, indicated significant improvement in patients’ condition relative to the baseline. This finding is in line with previous observational studies on minimally-invasive surgery for ULDH.^18^;^19^;^20^ conducted a follow-up of ULDH patients who underwent percutaneous endoscopic lumbar discectomy, and found an excellent rate of improvement in the short-term postoperative period (ca. 81.6%). A study by ^10^ showed that the frequency of outcomes rated as excellent or good according to the modified MacNab criteria was 96.43% at the last follow-up visit. In the present study, most patients had excellent or good outcomes according to the MacNab criteria (72.2%). The decrease in ODI, JOA, and VAS scores reflected a significant improvement in patient quality of life within 6 months of surgery.

However, there are some limitations to this study. Firstly, it was performed in a single center, which may limit the generalizability of the findings. Surgical protocols, patient demographic characteristics, and perioperative nursing practices may not be representative of the broader clinical setting. Secondly, the retrospective nature of the study has inherent limitations, including potential selection bias and incomplete data collection. Finally, the relatively small sample size limited the statistical efficacy of the analyses and may have compromised the identification of uncommon complications. Future studies should address these limitations using a multicenter, prospective design and a larger sample size. Long-term follow-up studies are also needed to assess the durability of UBE efficacy and the incidence of LDH recurrence.

## CONCLUSIONS

We showed that UBE technology has significant potential in the field of ULDH treatment; however, it is necessary to conduct large-scale clinical trials in the future to further explore its efficacy.
